# Consensus Diversity Plots: a global diversity analysis of chemical libraries

**DOI:** 10.1186/s13321-016-0176-9

**Published:** 2016-11-10

**Authors:** Mariana González-Medina, Fernando D. Prieto-Martínez, John R. Owen, José L. Medina-Franco

**Affiliations:** 1 Facultad de Química, Departamento de Farmacia, Universidad Nacional Autónoma de México, Avenida Universidad 3000, 04510 Mexico City, Mexico; 2 High-Performance Computing Research Group, ECIT Institute, Northern Ireland Science Park, Queens Road, Belfast, BT3 9DT UK

**Keywords:** Chemical space, Data mining, Molecular fingerprints, Molecular scaffolds, Physicochemical properties, Shannon entropy, Structural diversity

## Abstract

**Background:**

Measuring the structural diversity of compound databases is relevant in drug discovery and many other areas of chemistry. Since molecular diversity depends on molecular representation, comprehensive chemoinformatic analysis of the diversity of libraries uses multiple criteria. For instance, the diversity of the molecular libraries is typically evaluated employing molecular scaffolds, structural fingerprints, and physicochemical properties. However, the assessment with each criterion is analyzed independently and it is not straightforward to provide an evaluation of the “global diversity”.

**Results:**

Herein the Consensus Diversity Plot (CDP) is proposed as a novel method to represent in low dimensions the diversity of chemical libraries considering simultaneously multiple molecular representations. We illustrate the application of CDPs to classify eight compound data sets and two subsets with different sizes and compositions using molecular scaffolds, structural fingerprints, and physicochemical properties.

**Conclusions:**

CDPs are general data mining tools that represent in two-dimensions the global diversity of compound data sets using multiple metrics. These plots can be constructed using single or combined measures of diversity. An online version of the CDPs is freely available at: https://consensusdiversityplots-difacquim-unam.shinyapps.io/RscriptsCDPlots/.

**Graphical Abstract:**
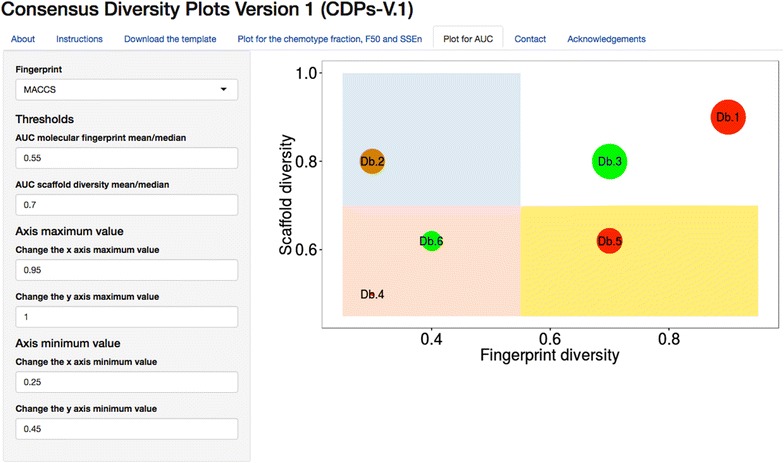
Consensus Diversity Plot is a novel data mining tool that represents in two-dimensions the global diversity of compound data sets using multiple metrics.

**Electronic supplementary material:**

The online version of this article (doi:10.1186/s13321-016-0176-9) contains supplementary material, which is available to authorized users.

## Background

Quantification of the chemical diversity in compound libraries is an important aspect in several areas of chemistry having a major impact in library acquisition, design, and selection for high-throughput screening (HTS) [1]. In drug discovery, assessment of the chemical diversity is crucial when it is desirable to explore novel regions of the medicinally relevant chemical space [2] or to keep the balance between diversity and novelty [3].

Multiple structural representations are needed for a comprehensive assessment of the diversity of compound libraries. There are several reports in which the diversity of compound data sets is analyzed using molecular scaffolds, structural fingerprints, and/or physicochemical properties [4–6]. This is because each representation has its own advantages and disadvantages: molecular scaffolds are straightforward to interpret but they capture part of the chemical structure, missing the information given by side chains [7]; structural fingerprints usually capture the information of the entire structure but are harder to interpret [8]. Whole molecular properties such as physicochemical properties are easy to interpret and are the basis of several drug- and lead-like empirical rules [9, 10]. However, these properties do not always distinguish the collections e.g., it is not uncommon for different compounds to share very similar property profiles. As such, considering multiple structure representations provides a broader picture of the diversity of the compounds libraries. However, the structural diversity calculated with each different criterion is analyzed independently and it is not straightforward to have a total perspective of the herein called “global diversity”.

As part of a continued effort to characterize the structural diversity of compound libraries in a combined manner, Consensus Diversity Plots (CDPs) are introduced in this work, representing a novel approach to compare the diversity of compound libraries considering three different structural representations simultaneously. To illustrate the application of CDPs the chemical diversity of eight compound databases and two subsets was analyzed using molecular scaffolds, structural fingerprints, and physicochemical properties. Scaffold diversity was assessed using counts, cyclic system recovery curves, and Shannon entropy (SE) [11] Fingerprint-based diversity was evaluated employing MACCS keys [12] and Extended Connectivity [13]/Tanimoto similarity, the diversity of physicochemical properties was assessed based on the Euclidean distance of the property profile of six properties frequently used in drug discovery [14, 15]. CDPs are general tools that can be built using other metrics and structural representations. Herein it is demonstrated that CDPs were able to differentiate the molecular libraries by global diversity.

## Methods

### Consensus Diversity Plot

**Fig. 1:**
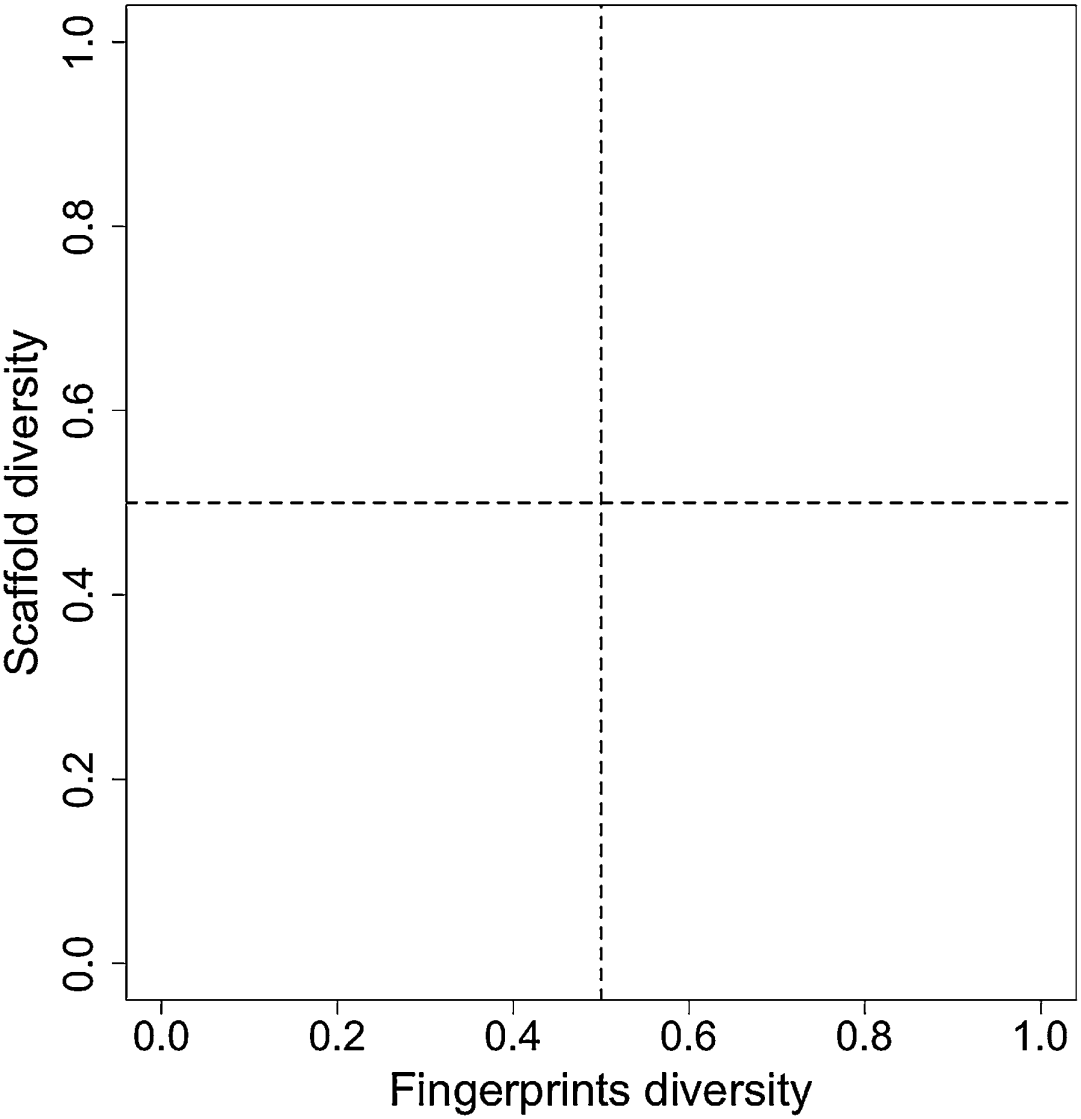
Prototype Consensus Diversity Plot. Scaffold diversity is plotted along the vertical axis and the fingerprints diversity is plotted along the horizontal axis. The thresholds (*dashed lines*) can be set depending on the metric used to quantify the diversity on each axis

Figure 1 depicts a prototype CDP. Scaffold diversity is plotted along the vertical axis and fingerprints diversity is plotted along the horizontal axis. A data point in the graph represents a compound data set. An additional diversity criterion such as physicochemical properties can be mapped in the CDPs with a continuous or categorical color scale. To aid in the interpretation of the plots, CDPs can be roughly divided into four quadrants (i.e., dashed lines) that classifies data sets as high/low diverse considering both, fingerprints and scaffolds. The scaffold diversity can be measured using metrics obtained from the cyclic system recovery (CSR) curves such as area under the curve (AUC), the fraction of scaffolds to retrieve 50% of the database (F_50_), or other metrics. As discussed in this manuscript, low AUC values point to high scaffold diversity whilst the opposite is true for F_50_ values.

For a broader comparison of the structural diversity of data sets, more than one plot can be generated using different metrics e.g., different measures of scaffold and fingerprint diversity. This would give rise to a series of CDPs that can be visually depicted in a single composite figure.

### Data sets

We compared data sets with different size (between 76 and 2500 compounds), types and source of molecules (e.g., small and large data sets, cyclic and acyclic; natural products and synthetic). The broad composition of the libraries was used to explore the ability of the CDPs to distinguish the compound collections using three criteria: molecular scaffolds, structural fingerprints, and physicochemical properties. Table 1 reports the number of unique compounds after data curation that was performed with the wash module of Molecular Operating Environment (MOE), version 2014.0 [16]. Data curation involved disconnecting metal salts, removing simple components, and rebalancing protonation states.

**Table 1:** Compound data sets analyzed in this work

Data set	Size^a^	Source
Natural products screening compounds (MEGx)	2500	ac-discovery.com
Semi-synthetic screening compounds (NATx)	2500	ac-discovery.com
Generally Recognized as Safe (GRAS)	2249	[17, 18]
GRAS subset (cyclic systems)	1195	[17, 18]
Carcinogenic	738	monographs.iarc.fr/ENG/Classification/index.php, [22]
Carcinogenic subset (cyclic systems)	544	monographs.iarc.fr/ENG/Classification/index.php, [22]
Anticancer drugs	76	[19]
Non-anticancer drugs	1399	[19]
Compounds in clinical trials (Clinical)	713	[21]
Epigenetic focused	850	selleckchem.com

^a^Number of unique compounds

Compound databases, shown in Table 1, included two commercial libraries from AnalytiCon, one with natural products containing mostly compounds derived from plants (MEGx), and the second with semi-synthetic molecules (NATx). These two collections are formatted for HTS, and reported as highly diverse by AnalytiCon. We also included 2249 compounds based on the Flavor and Extract Manufacturers Association of the United States—FEMA—GRAS list (hereafter referred to as ‘GRAS’) [17, 18]. Of note, GRAS compounds have broad applications in the food industry (and recently in drug discovery) but its diversity has been explored on a limited basis. Additional molecules were FDA drugs obtained from DrugBank Version 4.0 [19, 20]. These compounds were divided in 76 approved drugs to treat cancer (hereafter referred to as ‘anticancer drugs) and 1399 non-anticancer drugs. Other data sets analyzed were 713 compounds in clinical trials reported in the Therapeutic Target Database, TTD [21], 850 compounds from a commercial collection focused on epigenetic targets (hereafter referred to as ‘Epigenetic focused’), and 738 carcinogenic compounds reported by the International Agency for Research on Cancer—IARC—volumes 1–116, considering only substances classified as carcinogenic, probably and possibly carcinogenic (http://monographs.iarc.fr/ENG/Classification/index.php) and from the Carcinogenic Potency Database—CPDB [22].

### Molecular scaffolds and acyclic molecules

The term *scaffold* is used extensively to describe the core structure of a molecule. Different approaches to obtain the scaffold of a molecule in a consistent manner have been reviewed elsewhere [23, 24]. In this work, the scaffolds were derived with the methodology described by Johnson and Xu. The definition of scaffold used in this study is illustrated in Additional file 1: Figure S1. In this study, both acyclic and cyclic systems (hereafter referred to as chemotypes) were considered. However, to further characterize the behavior of the data sets containing more acyclic systems, GRAS and the Carcinogenic, the diversity of these data sets was also assessed removing acyclic systems. These subsets, hereafter referred to as *GRAS subset* and *Carcinogenic subset*, were compared to the data sets containing all the chemotypes (Table 1). The chemotypes were calculated with the program Molecular Equivalent Indices (MEQI) [25] and a code of five characters was assigned to each chemotype using a unique naming algorithm [26]. The program MEQI has been extensively used to conduct scaffold diversity analysis of compound databases [7, 27, 28].

#### Chemotype diversity

For each data set the number of chemotypes was recorded as well as the number of chemotypes containing only one compound i.e., singletons. The fraction of chemotypes and singletons relative to the number of molecules in the data set was computed.

#### Cyclic system retrieval (CSR) curves

CSR curves were plotted for each data set to analyze the distribution of the chemotypes [5]. To generate the CSR curves, the fraction of chemotypes is plotted on the X-axis and the fraction of compounds that contain those chemotypes is plotted on the Y-axis. CSR curves were further characterized calculating the values of AUC and F_50_. These metrics have been used to quantify scaffold diversity [11, 29].

#### Shannon entropy (SE)

The application of SE to design diverse chemical libraries and measure scaffold diversity has been reported [11]. In contrast to scaffold counts and CSR curves that consider the diversity of the entire library, SE can be focused on analyzing the diversity of a given *n* number of the most populated scaffolds [30]. The SE of a population of P compounds distributed in *n* systems is defined as:

1 $${\text{SE}} = - \mathop \sum \limits_{i = 1}^{n} p_{i} log_{2} p_{i}$$

2 $$p_{i} = \frac{{c_{i} }}{P}$$

where *p*_*i*_ is the estimated probability of the occurrence of a specific chemotype *i* in a population of *P* compounds containing a total of *n* acyclic and cyclic systems, and *c*_*i*_ is the number of molecules containing a particular chemotype. The value of SE ranges from 0, when all the compounds have the same chemotype (i.e., minimum diversity), to log_2_n, when all the compounds are evenly distributed among the *n* acyclic and/or cyclic systems (i.e., maximum diversity).

To normalize SE to the different *n* the scaled Shannon entropy (SSE) is defined as:

3 $${\text{SSE}} = \frac{SE}{{log_{2} n}}$$

Therefore, SSE values range from 0 (minimum diversity) to 1.0 (maximum diversity). To test the dependence of SSE with several maximum numbers of chemotypes, different numbers of *n* (e.g., 5–70) were considered. In a previous work a limited and arbitrary number of *n* most populated cyclic systems was explored [11].

### Structural fingerprints

For all pairs of compounds, the pairwise structural diversity was assessed with Molecular ACCess System (MACCS) keys (166-bits) [12] and Extended Connectivity Fingerprints (ECFP_4) [13] using the Tanimoto similarity coefficient [31]. The fingerprints were calculated with MayaChem Tools (http://www.mayachemtools.org/) and R Studio scripts [32]. MACCS keys/Tanimoto is a broadly used method to assess the diversity of compound data sets. However, CDPs can be generated using any other fingerprint representation or combination of them. Also, similarity coefficients other than Tanimoto [33] can be used.

### Physicochemical properties

Six properties of pharmaceutical relevance were calculated with MOE: hydrogen bond donors (HBD), hydrogen bond acceptors (HBA), the octanol and/or water partition coefficient (logP), molecular weight (MW), topological polar surface area (TPSA) and number of rotatable bonds (RTB). In MOE, the six properties have the following notation: a_don, a_acc, SlogP, Weight, TPSA, and b_rotN, respectively. These molecular descriptors have been used to measure the diversity of compound databases [34–36]. The distance (or *dissimilarity*) between any two data sets, *D*_*u*_ and *D*_*v*_, was computed using the Euclidean distance function [31] as follows. Let **x**_*i*_ be the N-dimensional vector of physicochemical properties for molecule *i* in dataset *D*_*u*_; similarly, let **y**_*j*_ be the N-dimensional vector of physicochemical properties for molecule *j* in dataset *D*_*v*_. (In the analyses of this article, six physicochemical properties were used so *N* = 6.) Let the number of molecules in data sets *D*_*u*_ and *D*_*v*_ be *U* and *V* respectively. Then the *inter*-data set distance $I_{uv}$ between data sets *D*_*u*_ and *D*_*v*_, as introduced in [37], was computed as:

$${I_{uv}} =  {1\over {UV}} {\sum ^U _{i=1}} {\sum ^V _{j=1}} {\text {euclidean}} \left( {{\textbf x}_{i} - {{\textbf y}}_{j} } \right),$$

$$\text {euclidean}\,({\textbf{x}}_i, {\textbf{y}}_j) = \sqrt{{\sum ^N _{k=1}}{(x_{ik} - y_{jk})}^2}.$$

In the special case when *u* = *v*, $I_{uv}$ is known as the *intra*-data set distance. Note that other sets of physicochemical properties can be used to produce CPDs. And also note that other distance functions can be used; comprehensive lists of distance functions are given in [38, 39].

## Results and discussion

As discussed in the “Methods” a key aspect of the CDPs is the quantification of the diversity of the data sets using different representations. In the following step, single metrics are selected to build the CDP (Fig. 1). This section is organized in four major parts: the first three show and discuss the results of the diversity of the eight compound databases and two subsets in terms of molecular scaffolds, fingerprints and physicochemical properties, respectively. The fourth section discusses the CDPs of the data sets and subsets.

### Diversity with molecular scaffolds

The scaffold diversity was assessed using frequency counts, CSR curves, and SSE.

#### Frequency counts

Table 2 summarizes the results of the scaffold counts, viz.; the number of chemotypes (N) in each set, the fraction of chemotypes relative to the number of molecules in each set (N/M) and the number and fraction of singletons (N_sing_).

**Table 2:** Summary of scaffold diversity analysis

Data set	N	N/M	N_sing_	N_sing_/N	N_sing_/M	AUC	F_50_
MEGx	935	0.374	642	0.687	0.257	0.781	0.072
NATx	799	0.320	400	0.501	0.160	0.768	0.116
GRAS	238	0.106	150	0.630	0.067	0.926	0.004
GRAS subset	237	0.198	150	0.633	0.126	0.867	0.021
Carcinogenic	262	0.355	195	0.744	0.264	0.800	0.031
Carcinogenic subset	261	0.480	195	0.747	0.450	0.737	0.107
Anticancer drugs	70	0.921	65	0.929	0.855	0.537	0.457
Non-anticancer drugs	844	0.572	686	0.813	0.465	0.699	0.157
Clinical	603	0.846	565	0.937	0.792	0.576	0.409
Epigenetic focused	727	0.855	666	0.916	0.784	0.569	0.415

*N* number of chemotypes, *M* number of molecules, *N*_*sing*_ number of singletons, *AUC* area under the curve, *F*_*50*_ fraction of chemotypes that contains 50% of the data set

The data sets containing drugs approved by the FDA to treat cancer, Clinical and Epigenetic focused had the largest proportion of chemotypes relative to the number of molecules (N/M) and the largest proportion of singletons relative to the number of molecules (N_sing_/M), greater than 0.84 and 0.78, respectively. Interestingly, the library with approved drugs to treat cancer, which has the lowest number of compounds (78, see Table 1), is the most diverse considering the proportion of singletons relative to the number of molecules (N_sing_/M = 0.855) and the number of chemotypes relative to the number of compounds (N/M = 0.921). Surprisingly, GRAS, NATx, MEGx, the largest data sets (with more than 2000 compounds), had the lowest scaffold diversity as measured by the small proportion of singletons relative to the total number of scaffolds (N_sing_/N lower than 0.69) and relative to the total number of molecules (N_sing_/M lower than 0.26). Similar trends can be deduced from the fraction of scaffolds relative to the total number of molecules (N/M) summarized in Table 2. As indicated in the “Methods” section, GRAS is a large collection of compounds used in the food industry; NATx and MEGx are commercial data sets of natural products (available for HTS). As expected, after removing all the acyclic systems from GRAS and the carcinogenic data sets, their N/M and N_sing_/M increased (Table 2). However, comparing the diversity of all the data sets the relative diversity order did not change decreasing in the following order: anticancer drugs > epigenetic focused > clinical > non-anticancer drugs > carcinogenic subset > carcinogenic > MEGx > NATx > GRAS subset > GRAS. Based on the scaffold results, GRAS would be the least diverse data set.

#### CSR curves

As explained in detail elsewhere [36] CSR curves represent the fraction of molecules in the data set contained in a fraction of chemotypes. A data set with maximum diversity would contain a different chemotype for each molecule in the library and the curve would be a diagonal with AUC of 0.5. As the scaffold diversity decreases the curve will move away from the diagonal. The minimum diversity would be a data set where all the compounds have the same chemotype. In this case, the CSR would be a vertical line with AUC equal to 1.0.

**Fig. 2:**
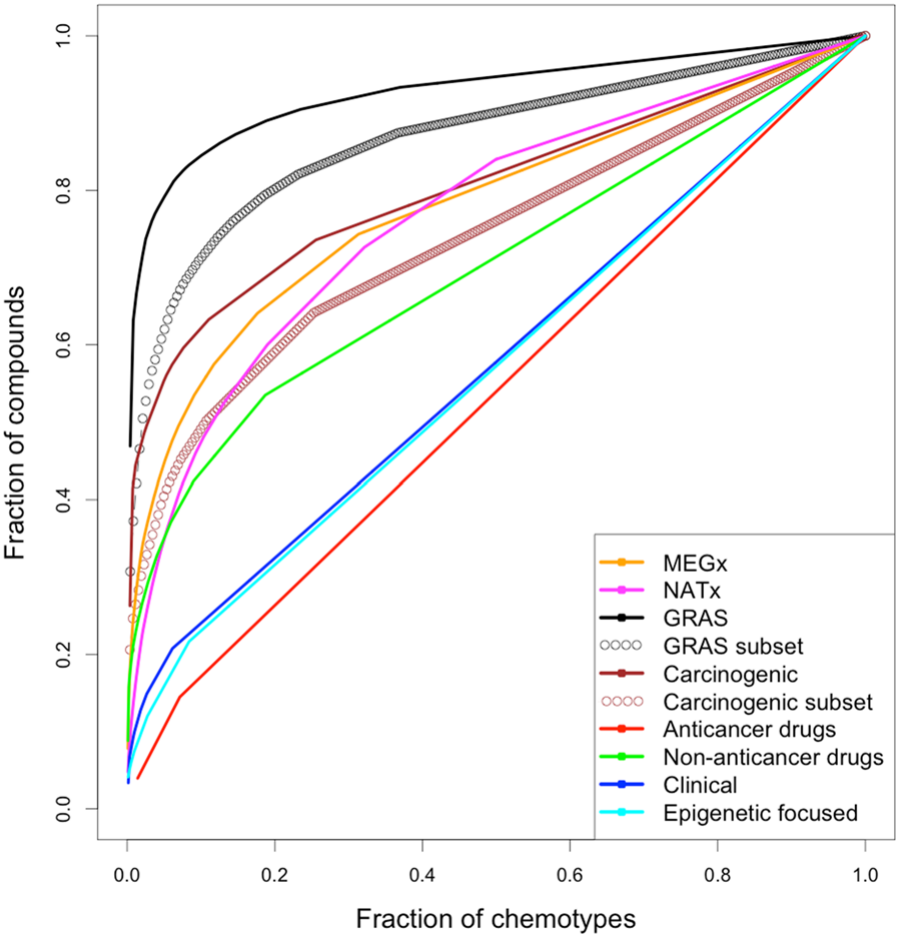
Scaffold retrieval curves (CSR) curves for the data sets studied in this work. Area under the curve (AUC) and fraction of chemotypes required for retrieving 50% of the compounds in the data sets (F_50_) are summarized in Table 2

Figure 2 shows the CSR curves for the data sets and subsets. The curves for the approved anticancer drugs, Clinical and Epigenetic focused indicate large diversity e.g., curves close to the diagonal. In contrast, the curves for GRAS and Carcinogenic, followed by MEGx and NATx, indicate lower diversity. The curves for GRAS and carcinogenic improved significantly after removing the acyclic systems, particularly for Carcinogenic.

Table 2 summarizes the AUC and F_50_ values that were used to compare the curves for each set quantitatively. Consistent with the shape of the curves, anticancer drugs, Clinical and Epigenetic focused showed the lowest AUC values (lower than 0.580) and also the largest F_50_ values (close or larger than 0.409). High F_50_ values mean that 50% of the compounds in the data sets are contained in a large number of scaffolds: for instance, 50% of the anticancer drugs were distributed in 46% of the scaffolds (Table 2). For comparison, 50% of GRAS compounds (the least diverse set) were contained in 0.4% of the total scaffolds.

According to the AUC and F_50_ values as measures of scaffold diversity, GRAS followed by Carcinogenic were the least diverse (Table 2). The low diversity of GRAS and Carcinogenic data sets is due to that 46.9 and 26.3% of the molecules in these data sets, respectively, share the same chemotype. The AUC and F_50_ for the GRAS subset were better than the AUC and F_50_ calculated for GRAS, which includes acyclic and cyclic systems; yet after removing the acyclic systems this data set is still the least diverse by scaffolds. When it comes to the Carcinogenic subset, its AUC and F_50_ improved, making it change from being one of the least diverse data sets to becoming moderately diverse by scaffolds.

Overall, the scaffold-diversity assessments measured with scaffold counts and CSR curves were in agreement showing that the anticancer drugs data set was the most diverse (despite it being the smallest data set), whilst the GRAS and GRAS subset data sets were the least diverse.

#### Scaled Shannon entropy

**Fig. 3:**
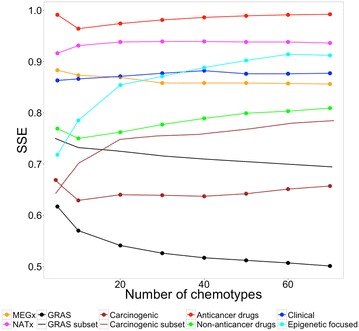
Results of scaled Shannon entropy (SSE) at different numbers of most populated chemotypes

CSR curves are useful to compare the chemotype diversity of data sets, however, they do not provide information concerning the distribution of compounds in each chemotype or in the most populated chemotypes [11]. The distribution of the compounds in the most populated chemotypes was analyzed with the SSE as described in “Methods”. For all the sets, we studied up to the first 70 most populated chemotypes considering the total number of chemotypes in the data set with approved drugs to treat cancer, which was the most diverse set according to chemotype counts and CSR curves. SSE is a measure of the specific distribution of molecules in a given number of chemotypes. A small value of SSE indicates that the molecules are distributed in a small number of different chemotypes (lower diversity). A value of SSE closer to one indicates that the molecules are evenly distributed in the different chemotypes (higher diversity). Figure 3 summarizes the results of SSE at different numbers of the most populated chemotypes. The values of each data set are in the Additional file 1: Table S1.

Figure 3 reveals that there was not a dramatic dependence of SSE with the number of *n* most populated scaffolds. Taking together all the SSE values at different number of scaffolds (SSE5–SSE70) it can be concluded that the scaffold diversity of the eight data sets, as captured by SSE, decreases in the order: anticancer drugs > NATx > Epigenetic focused > Clinical > MEGx > non-anticancer drugs > Carcinogenic subset > GRAS subset > Carcinogenic > GRAS. The diversity of GRAS and Carcinogenic did not change after removing the acyclic systems from the data sets. This relative order of diversity is in general agreement the rank-ordering obtained with other measures of scaffold diversity discussed in previous sections. A notable exception, however, is the scaffold diversity of NATx: a data set of natural products that shows a relative high diversity only when the most populated cyclic systems are considered as is the case of SSE. Of note, as discussed in previous sections, NATx has a relative low scaffold diversity considering metrics from the CSR curves. In some cases, the most populated scaffolds in a data set are a representative sample of the composition of the database being studied.

The lower diversity of the approved non-anticancer drugs compared to the approved drugs to treat cancer is an unexpected finding. It would be expected to find many more molecules with different chemotypes in a data set that contains drugs for different therapeutic uses.

#### Diversity with structural fingerprints

As pointed out in the Introduction, analysis of the scaffold diversity does not include information on the diversity of the compounds associated with the side chains. A good example is the GRAS data set where nearly half of the compounds have acyclic molecules. Therefore, other methods are required to complement the assessment of the global diversity. One of such methods is the fingerprint-based diversity that provides information on the diversity of the entire molecule. To illustrate this point, MACCs keys (166-bits) fingerprints, and ECFP were computed to determine the intermolecular diversity of the libraries. Other fingerprint-based representations can be used. In this work we employed MACCS keys/Tanimoto (see “Methods”) because it is a well-known and broadly used representation to quantify structural diversity. Table 3 summarizes the distributions of the intra-library similarity results obtained with MACCS keys and Additional file 1: Table S2 summarizes the similarity results for ECFP. The compound data sets with the highest structural diversity (e.g., smaller similarity values) were, in general, the carcinogenic compounds (e.g., median MACCS keys/Tanimoto similarity of 0.229) followed by GRAS. Interestingly, these libraries were the least diverse when studied based solely on the chemotypes and their similarity values increased after removing the acyclic systems, which means acyclic systems contribute to the diversity of these data sets. The large difference between scaffold and fingerprint-based similarity for these libraries is because a large number of compounds share the same chemotype. Also remarkable was the high fingerprint-based diversity of the approved non-anticancer drugs (e.g., median MACCS keys/Tanimoto similarity close to 0.370), that was less diverse than the anticancer drugs considering its scaffold diversity (Table 2; Fig. 2), and the high similarity values for the anticancer drugs (median MACCS keys/Tanimoto similarity close to 0.468) classified as the second least diverse data set with fingerprints but the most diverse data set by content of chemotypes. These results clearly illustrate the large influence of the side chains and acyclic systems on the assessment of the global diversity and how different the diversity results can be depending on the method employed, making it hard to classify different data sets by their diversity analyzing the results of each metric separately.

**Table 3:** Summary of the intra-library similarity distributions computed with MACCS keys/Tanimoto

Data set	Min.	1st Qu.	Median	Mean	3rd Qu.	Max.	SD
MEGx	0	0.388	0.475	0.485	0.574	1.0	0.138
NATx	0.104	0.378	0.444	0.460	0.525	1.0	0.119
GRAS	0	0.256	0.375	0.385	0.500	1.0	0.181
GRAS subset	0.016	0.269	0.38	0.385	0.487	1.0	0.161
Carcinogenic	0	0.135	0.229	0.244	0.333	1.0	0.151
Carcinogenic subset	0.014	0.180	0.269	0.284	0.370	1.0	0.144
Anticancer drugs	0.065	0.362	0.468	0.460	0.562	1.0	0.139
Non-anticancer drugs	0	0.283	0.370	0.373	0.458	1.0	0.130
Clinical	0	0.345	0.438	0.432	0.522	1.0	0.128
Epigenetic focused	0.039	0.344	0.430	0.431	0.516	1.0	0.123

*1st Qu.* first quartile, *3rd Qu.* third quartile

The results obtained with ECFP4 (Additional file 1: Table S2), demonstrate that this fingerprint was not helpful to identify and classify data sets by their structural diversity, giving comparable similarity values. Even though ECFP4 have demonstrated to perform well for virtual screening [40], MACCS keys fingerprints performed better differentiating the data sets.

### Diversity with physicochemical properties

Table 4 shows the mean of all the pairwise Euclidean distances of the six physicochemical properties for all the compounds within each data set (e.g., intra-set distance). Other metrics such as the median values could be explored. According to these values, the most diverse sets were MEGx and anticancer drugs (mean Euclidean distance of 2.9) suggesting that these collections have, in general, a broad distribution of the six physicochemical properties. Of note, the anticancer set also had the largest scaffold diversity.

**Table 4:** Mean of the intra-set Euclidean distance of six physicochemical properties

Data set	Euclidean distance
MEGx	2.95
NATx	1.07
GRAS	1.00
Carcinogenic	2.22
Anticancer drugs	2.90
Non-anticancer drugs	2.50
Clinical	2.62
Epigenetic focused	2.10

### CDPs of the test compound collections

**Fig. 4:**
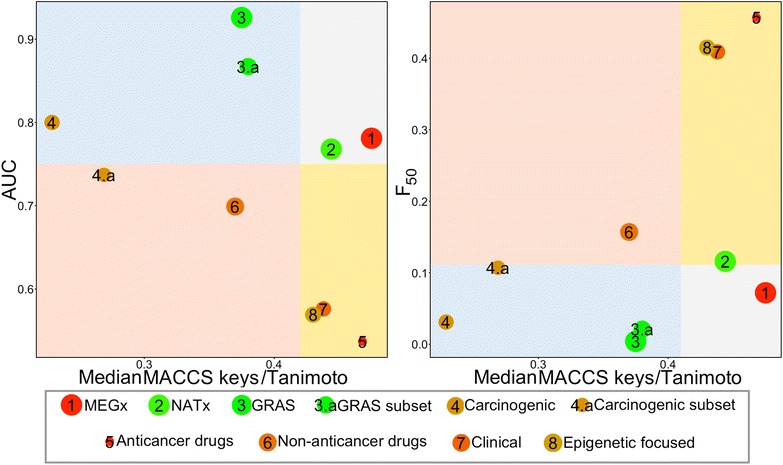
Consensus Diversity Plots (CDPs) for the eight data sets and two subsets studied in this work. CDPs in this figure classify the compound data sets considering molecular scaffolds, fingerprint representations, and physicochemical properties. Each data point represents a compound set. Fingerprint-based diversity is plotted on the *X*-axis. Scaffold diversity is represented in the *Y*-axis plotting area under the curve (AUC) or F_50_. The quadrants in *red* identify compound data sets with high fingerprint-based diversity, the quadrants in *white* identify data sets with relative low fingerprint-based diversity and lower scaffold diversity; the quadrants in *blue* locate data sets with high fingerprint-based diversity but low scaffold diversity; and the quadrants in *yellow* identify compound libraries with low fingerprint-based diversity but high scaffold diversity. Data points are *colored* by the diversity of the physicochemical properties of the data set as measured by the Euclidean distance of six properties of pharmaceutical relevance. The distance is represented with a continuous color scale from *red* (more diversity), to *orange*/*brown* (intermediate diversity) to *green* (less diversity). The relative size of the data set is represented with the size of the data point: smaller data points indicate compound data sets with fewer molecules. In this application example of the plots, a value of 0.75 for AUC and the median values of the distribution of F_50_ and MACCS/Tanimoto similarity were used to set the quadrants

From the analysis discussed so far it is not obvious to identify the data set with the overall highest scaffold, fingerprint-based, and physicochemical properties diversity. To address this issue, Fig. 4 shows CDPs representing the diversity of eight compound data sets and two subsets studied in this work. CDPs portray the diversity calculated with three major molecular representations: molecular scaffolds, fingerprints, and physicochemical properties. Each data point represents one data set.

Diversity based on fingerprints and molecular scaffolds is represented on the X- and Y-axis, respectively. Fingerprint-based diversity is represented in these plots with the corresponding median of the MACCS keys/Tanimoto similarity of the data set. In each CDP the scaffold diversity of the entire collection is represented on the Y-axis using AUC or F_50_, respectively.

In these plots, diversity based on physicochemical properties is represented by the intra-set Euclidean distance of the six physicochemical properties i.e., values in Table 4. This value is represented in the CDPs using a continuous color scale from red (high distance; high diversity), to orange/brown (intermediate diversity), to green (low distance; low diversity). The relative size of the data set is represented by the size of the data point: bigger points correspond to databases with more compounds (Table 1).

To assist in the classification of the compound data sets by their global diversity, four major regions were identified in the CDPs using heuristic thresholds along the X- and Y-axis, respectively. In this work, the thresholds were defined by the median values of the distribution of the MACCS keys/Tanimoto similarity and AUC value of 0.75 (as detailed in “Methods”). As per F_50_, we also used the corresponding median value of all the sets (0.111). We would like to point out that other values can be explored to define the thresholds. Moreover, as discussed in “Methods” (prototype plot in Fig. 1), these plots are useful even in the absence of thresholds: the most relevant feature of the CDPs is that they enable to visually represent the diversity of compound data sets in low dimensions.

The quadrants in red, Fig. 4 and Additional file 1: Figure S3, identify compound data sets with high fingerprint-based diversity i.e., low median MACCS and ECFP4/Tanimoto similarity and high scaffold diversity e.g., low AUC or high F_50_. The quadrants in white identify data sets with relative low fingerprint-based diversity and lower scaffold diversity. In other words, data sets in the red or white quadrants are collections with the relative highest or lowest, respectively, fingerprint-based and scaffold diversity combined. The quadrants in blue locate data sets with high fingerprint-based diversity but low scaffold diversity. This means that the structural diversity of the compound data sets located in this region is driven mostly by the side chains or, as it was observed with the subsets from GRAS and Carcinogenic, by the acyclic systems. Finally, the quadrants in yellow identify compound libraries with low fingerprint-based diversity but high scaffold diversity. In this case, the major contributors to the diversity are the molecular scaffolds (or the proportion of acyclic systems present). Of note, the diversity in terms of physicochemical properties and the size of the library do not determine the position of the data sets in the quadrants but provide additional information.

Since three types of diversity (based on scaffolds, fingerprints and properties) are represented in the CPDs with a single value each, these plots do not capture the entire profile of, for example, scaffold diversity. In other words, the visual representation of several different types of diversity in a single plot (that is the high value of the CDPs) comes with the cost that some information is lost.

According to the CDPs in Fig. 4, non-anticancer drugs are the most diverse set considering MACCS keys and chemotypes when metrics of scaffold diversity of the entire collection are considered (AUC and F_50_). Anticancer drugs have an intermediate diversity considering the physicochemical properties (orange-to-red data point). In contrast, the natural product data sets MEGx and NATx are, relative to the other data sets, the least diverse considering MACCS keys and molecular scaffolds. Of note, these two data sets have the largest number of compounds as depicted by the relative bigger size of the data points. These results further illustrate that the size of the library is not necessarily correlated with the structural diversity [11].

CDPs in Fig. 4 visually emphasize that despite the fact that MEGx and NATx sets have similar values of fingerprint-based and scaffold diversity, they have very different diversity in terms of physicochemical properties: MEGx (data point in red) is one of the most diverse sets whilst NATx (data point in green) is one of the least diverse. By analogy with the concept of activity [41] and property cliffs [42] this observation could be associated with a *multiple*-*diversity cliff*: compound collections that share similar diversity according to molecular representations (e.g., scaffold and fingerprints) but have opposite diversity considering other representation (e.g., physicochemical properties). The quite different diversity profile of MEGx and NATx is in agreement with the notion that synthetic and semi-synthetic compounds (NATx) are, on average, less diverse than natural products (MEGx). This is because, in general, semi-synthetic compounds such as NATx are designed within the constraints of drug-like empirical rules.

The relative position of GRAS and Carcinogenic sets in the CDPs (quadrant in blue) indicates that both compound collections have relative low MACCs keys/Tanimoto similarity but low chemotype diversity (e.g., high AUC or low F_50_ values). Indeed, as discussed previously in this study, GRAS and Carcinogenic have a large proportion of molecules with a single chemotype, i.e., acyclic systems. After removing the acyclic systems from GRAS the scaffold diversity of the GRAS subset did not change significantly, however the structural diversity as measured with MACCS keys/Tanimoto decreased. Something similar occurred with the carcinogenic subset, after removing the acyclic systems the similarity increased, however the scaffold diversity increased, therefore the subset was classified as diverse by scaffolds and fingerprints. These results suggest structural diversity in the blue quadrant, as measured with MACCS keys/Tanimoto, is also influenced by the acyclic systems. Considering physicochemical properties, both sets have a different diversity profile: GRAS (data point in green) has low diversity while Carcinogenic (data point in brown) has higher diversity.

Approved anticancer drugs, Epigenetic focused and Clinical (in quadrant in yellow) are sets with relative low MACCs keys/Tanimoto similarity but high scaffold diversity (e.g., low AUC or high F_50_ values). This classification suggests that the diversity of the compound data sets is driven by the scaffolds. The large scaffold diversity of anticancer drugs contrasts with the relative small size of the data set. This result further emphasizes the convenience of using multiple features of the data sets to have a better and quick assessment of the global (or consensus) diversity. In addition, CDPs in Fig. 3 emphasize that approved anticancer and non-anticancer drugs have different profiles of diversity, including physicochemical properties. Putting together these observations with previous analyses comparing approved anticancer and non-anticancer drugs [34] confirm that concepts such as “drug-likeness” highly depend on the type of drugs being analyzed.

Additional file 1: Figure S2 shows CDPs using ECFP4 to measure fingerprint diversity. As it can be observed on these plots this fingerprint was unable to differentiate the diversity of anticancer drugs, Clinical and Epigenetic focused data sets. Therefore this metric was not chosen to classify these data sets. Of note, CDPs can be built using other fingerprints or combination of them, depending on the data sets being analyzed.

#### CDPs based on scaffold diversity focused on the most populated cyclic systems

We further explored the representation of the global diversity of the data sets generating CDPs using measures of scaffold diversity based on the most populated scaffolds e.g., SSE5 and SSE70. The plots are shown in Additional file 1: Figure S3. To generate the quadrants in these plots the median of the distribution of the SSE5 and SSE70 were used (0.758 and 0.833, respectively). In general, the relative classification of the eight data sets was comparable to the CDPs using the metrics AUC and F_50_. One of the few but notable differences was the relative classification of approved non-anticancer drugs (changing its position from the red to the blue quadrant), as it was observed before this data set is not one of the most diverse by scaffolds (Table 2) but it is diverse by fingerprints (Table 3). Therefore, this data set diversity could be given mostly by its side chains and it can be found on the blue or red quadrant depending on the scaffold metric employed. The second difference was the classification of NATx modifying its position in the CDPs from the white to the yellow quadrant. This modification is due to these data sets different scaffold diversity considering only the 5 or up to 70 of the most populated scaffolds as compared to the diversity of the entire collections. All other six data sets kept the same relative position in the CDPs. These results indicate that different measures of scaffold diversity can be used in a CDP. The most appropriate measure will depend on the specific goals of the project.

As discussed throughout the manuscript CDPs provide a comprehensive representation of the structural diversity of the compound data sets, despite the fact that these plots do not provide inter-library relationship. However, such analysis can be conducted separately using other established approaches [43, 44].

## Conclusions

Consensus Diversity Plots (CDPs) are data mining tools that represent in two-dimensions the global diversity of compound data sets using multiple metrics. CDPs are useful to compare and classify chemical libraries. The specific applications depend on goals of the study. For example, to identify new hits it is desirable to screen diverse libraries. According to the similarity principle, to optimize hits might be preferable to screen focused libraries that resemble the structure of the hit compounds. Herein was shown the application of CDPs to represent the global diversity of eight test data sets. Three features frequently used to characterize the diversity of chemical libraries were considered: molecular scaffolds, fingerprints, and physicochemical properties. In addition, the size of the data sets was mapped in the plots. CDPs are general and can be constructed using any molecular similarity method or scaffold diversity metric. A limitation of the current version of these plots, in particular the definition of thresholds for high/low scaffold or fingerprint-based diversity, is that they depend on the data sets being compared. Of the test data sets analyzed in this work, CDPs identified the approved non-anticancer drugs as the set with the highest combined scaffold and MACCS keys/Tanimoto similarity. The diversity profile of this set is considerably different from the global diversity of the approved anticancer drugs. Surprisingly, CDPs also identified two large data sets of natural products commercially available (derived from plants and semi-synthetic compounds, respectively) as the least diverse sets relative to the other six compound collections. Moreover, CDPs visually depicted the dramatic difference in the diversity of the physicochemical properties of these two data sets despite the fact that the two natural product collections have similar profiles of scaffold and fingerprint-based diversity. CDPs also identified GRAS and carcinogenic as compound sets with low scaffold diversity but large fingerprint-based diversity indicating that the diversity is mainly associated with the acyclic systems. Similarly, there were data sets with high scaffold-diversity but relatively low fingerprint-based diversity indicating that the diversity of such data sets (e.g., approved anticancer drugs) is due mostly to the molecular scaffolds. Finally, CDPs further illustrated that the size of the data set is not necessarily associated with the global diversity.

Since CDPs are general tools other meaningful similarity methods can be used, for instance, consensus measures of fingerprint-based similarity [45]. In addition, depending on the goals of the project, different thresholds can be implemented to define high/low fingerprint-based diversity and scaffold diversity. A major perspective of this work is to develop a consensus measure of global diversity. Efforts in this direction are being undertaken in our research group.

## Additional file

**Additional file 1: Table S1.** Scaffold diversity using scaled Shannon entropy at different numbers of most populated scaffolds.** Table S2.** Summary of the intra-library similarity distributions computed with Extended Connectivity/Tanimoto.** Figure S1.** Definition of molecular scaffold used in this work.** Figure S2.** Consensus Diversity Plots for the eight data sets and two subsets studied in this work using Extended Connectivity/Tanimoto.** Figure S3.** Consensus Diversity Plots using measures of scaffold diversity for the most populated scaffolds.

## Abbreviations

AUC: area under the curve
CDP: Consensus Diversity Plot
CPDB: Carcinogenic Potency Database
CSR curves: cyclic systems retrieval curves
F_50_: fraction of chemotypes required to retrieve 50% of the compounds in each database
FDA: Food and Drug Administration
GRAS: Generally Recognized as Safe
HBA: hydrogen bond acceptors
HBD: hydrogen bond donors
HTS: high-throughput screening
IARC: International Agency for Research on Cancer
LogP: the octanol and/or water partition coefficient
MEQI: Molecular Equivalent Indices
MOE: Molecular Operating Environment
MW: molecular weight
RTB: rotatable bonds
SE: Shannon entropy
SSE: scaled Shannon entropy
TPSA: topological polar surface area
TTD: Therapeutic Target Database
