# Supplementary material for: Consensus Diversity Plots: a global diversity analysis of chemical libraries
Source: J Cheminform. 2016 Nov 10;8:63. doi: 10.1186/s13321-016-0176-9 (PMC5105260; doi:10.1186/s13321-016-0176-9)
Supplement: Supplementary file 1 — Additional file 1: Table S1. Scaffold diversity using scaled Shannon entropy at different numbers of most populated scaffolds. Table S2. Summary of the intra-library similarity distributions computed with Extended Connectivity/Tanimoto. Figure S1. Definition of molecular scaffold used in this work. Figure S2. Consensus Diversity Plots for the eight data sets and two subsets studied in this work using Extended Connectivity/Tanimoto. Figure S3. Consensus Diversity Plots using measures of scaffold diversity for the most populated scaffolds. [file 13321_2016_176_MOESM1_ESM.pdf]

Additional File 1

Consensus Diversity Plots: A Global Diversity Analysis of Chemical Libraries

Mariana González-Medina,[1] Fernando D. Prieto-Martínez,[1] John R. Owen,[2] José L. Medina-Franco[1*]

[1]*Facultad de Química, Departamento de Farmacia, Universidad Nacional Autónoma de México, Avenida Universidad 3000, Mexico City 04510, Mexico*

[2]*High-Performance Computing Research Group, ECIT Institute, Northern Ireland Science Park, Queens Road, Belfast BT3 9DT, United Kingdom*

Table S1. Scaffold diversity using scaled Shannon entropy (SSE) at different numbers of most populated scaffolds

Data set	SSE5	SSE10	SSE20	SSE30	SSE40	SSE50	SSE60	SSE70
MEGx	0.883	0.873	0.869	0.858	0.858	0.858	0.857	0.856
NATx	0.916	0.931	0.938	0.939	0.939	0.938	0.938	0.936
GRAS	0.617	0.57	0.541	0.526	0.517	0.512	0.507	0.501
GRAS subset	0.748	0.732	0.725	0.716	0.710	0.705	0.700	0.695
Carcinogenic	0.664	0.629	0.64	0.639	0.637	0.642	0.651	0.657
Carcinogenic subset	0.647	0.701	0.748	0.756	0.759	0.768	0.779	0.784
Anticancer drugs	0.991	0.964	0.974	0.981	0.986	0.989	0.991	0.992
Non-anticancer drugs	0.769	0.75	0.762	0.777	0.789	0.799	0.803	0.809
Clinical	0.863	0.866	0.871	0.877	0.882	0.876	0.876	0.877
Epigenetic focused	0.718	0.785	0.854	0.871	0.888	0.902	0.914	0.912

SSE:Scaled Shannon Entropy at the 70 most populated chemotypes

Table S2. Summary of the intra-library similarity distributions computed with Extended Connectivity (ECFP_4)/ Tanimoto

Data set	Min.	1st Qu.	Median	Mean	3rd Qu.	Max.	SD
MEGx	0.000	0.101	0.128	0.135	0.160	1.000	0.052
NATx	0.013	0.111	0.136	0.150	0.167	1.000	0.072
GRAS (cyclic and aclycic systems)	0.000	0.091	0.130	0.141	0.178	1.000	0.073
GRAS subset (cyclic systems)	0.000	0.095	0.131	0.139	0.171	1.000	0.067
Carcinogenic	0.000	0.050	0.081	0.089	0.116	1.000	0.059
Carcinogenic subset(cyclic systems)	0.000	0.062	0.091	0.098	0.125	1.000	0.057
Anticancer drugs	0.014	0.095	0.120	0.123	0.146	0.832	0.053
Non-anticancer drugs	0.000	0.088	0.114	0.117	0.141	1.000	0.046
Clinical	0.000	0.098	0.122	0.122	0.146	0.797	0.039
Epigenetic focused	0.000	0.100	0.123	0.126	0.149	1.000	0.040

1stQ: first quartile; 3rd Q: third quartile

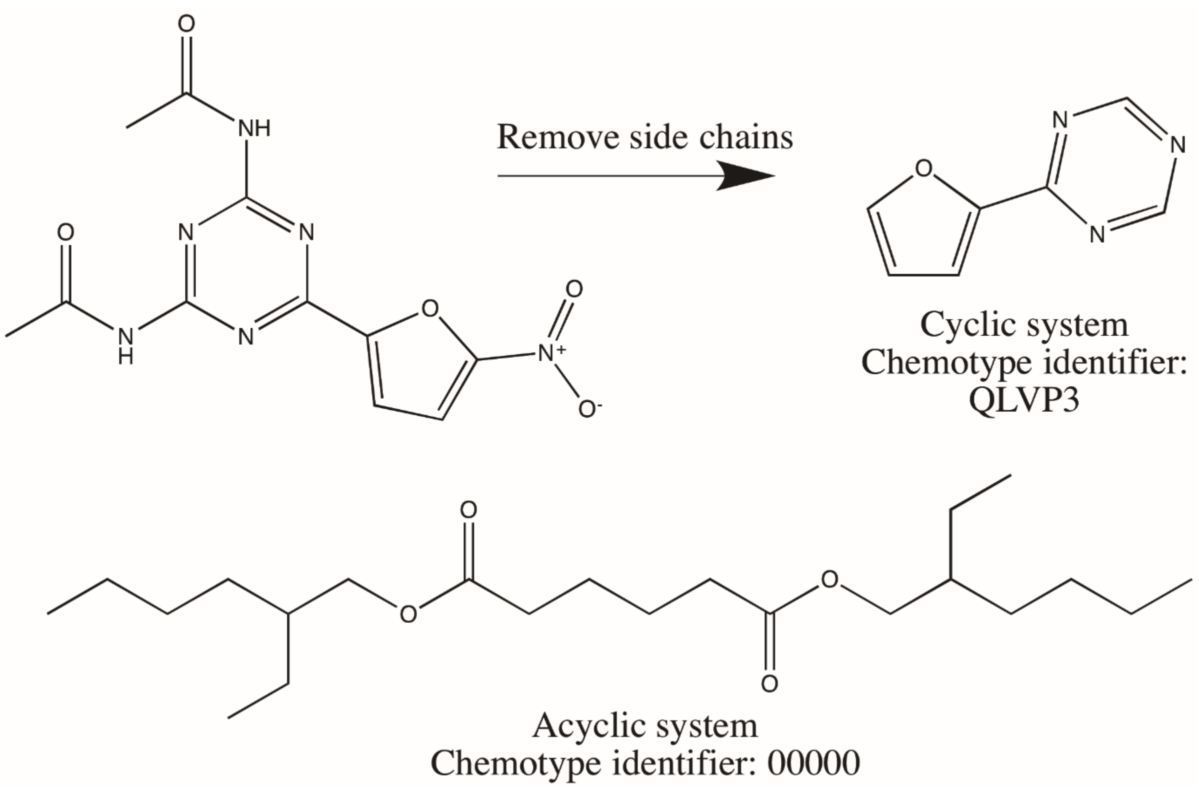

Figure S1. Definition of molecular scaffold used in this work. The scaffold (*or* cyclic system) was obtained after iteratively removing the side chains of the entire molecule. Heteroatoms in the scaffold are considered part of the scaffold. Each cyclic system is assigned with a code (*i.e.*, chemotype identifier) of five characters following the approach of Johnson and Xu (Xu Y-J, Johnson M. Algorithm for naming molecular equivalence classes represented by labeled pseudographs. J Chem Inform Comput Sci. 2001;41(1):181-5). In this approach, acyclic systems are assigned with the chemotype identifier ´00000´.

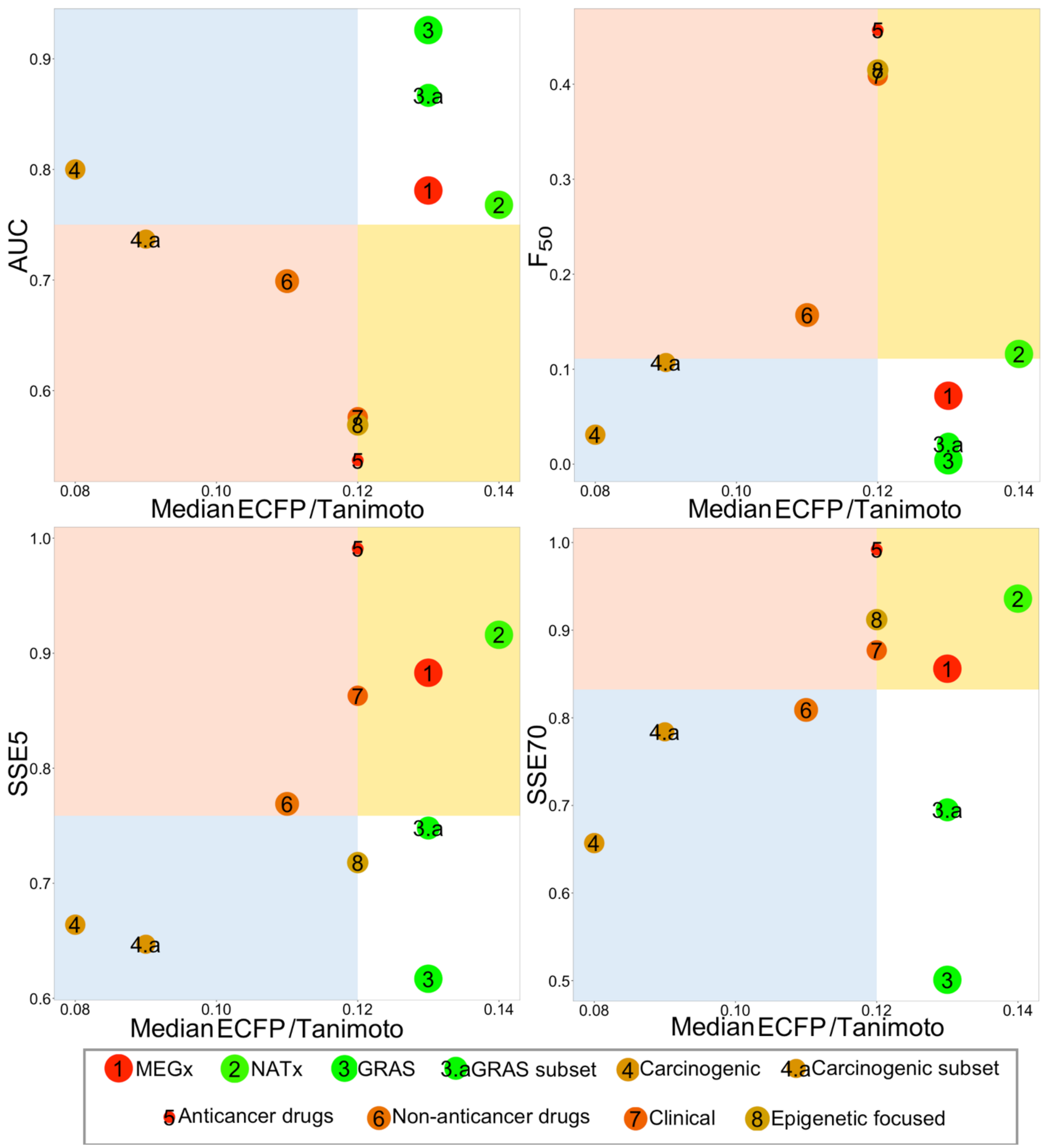

Figure S2. Consensus Diversity Plots (CDPs) for the eight data sets and two subsets studied in this work. CDPs in this figure classify the compound data sets considering molecular scaffolds, fingerprint representations, and physicochemical properties. Each data point represents a compound set. Fingerprint-based diversity is plotted on the X-axis. Scaffold diversity is represented in the Y-axis plotting area under the curve (AUC), F_{50}, SSE5 and SSE70. The quadrants in red identify compound data sets with high fingerprint-based diversity, the quadrants in white identify data sets with relative low fingerprint-based diversity and lower scaffold diversity; the quadrants in blue locate data sets with high fingerprint-based

diversity but low scaffold diversity; and the quadrants in yellow identify compound libraries with low fingerprint-based diversity but high scaffold diversity. Data points are colored by the diversity of the physicochemical properties of the data set as measured by the Euclidean distance of six properties of pharmaceutical relevance. The distance is represented with a continuous color scale from red (more diversity), to orange/brown (intermediate diversity) to green (less diversity). The relative size of the data set is represented with the size of the data point: smaller data points indicate compound data sets with fewer molecules. In this application example of the plots, a value of 0.75 for AUC and the median values of the distribution of F_{50}, SSE5, SSE70 and ECFP/Tanimoto similarity were used to set the quadrants.

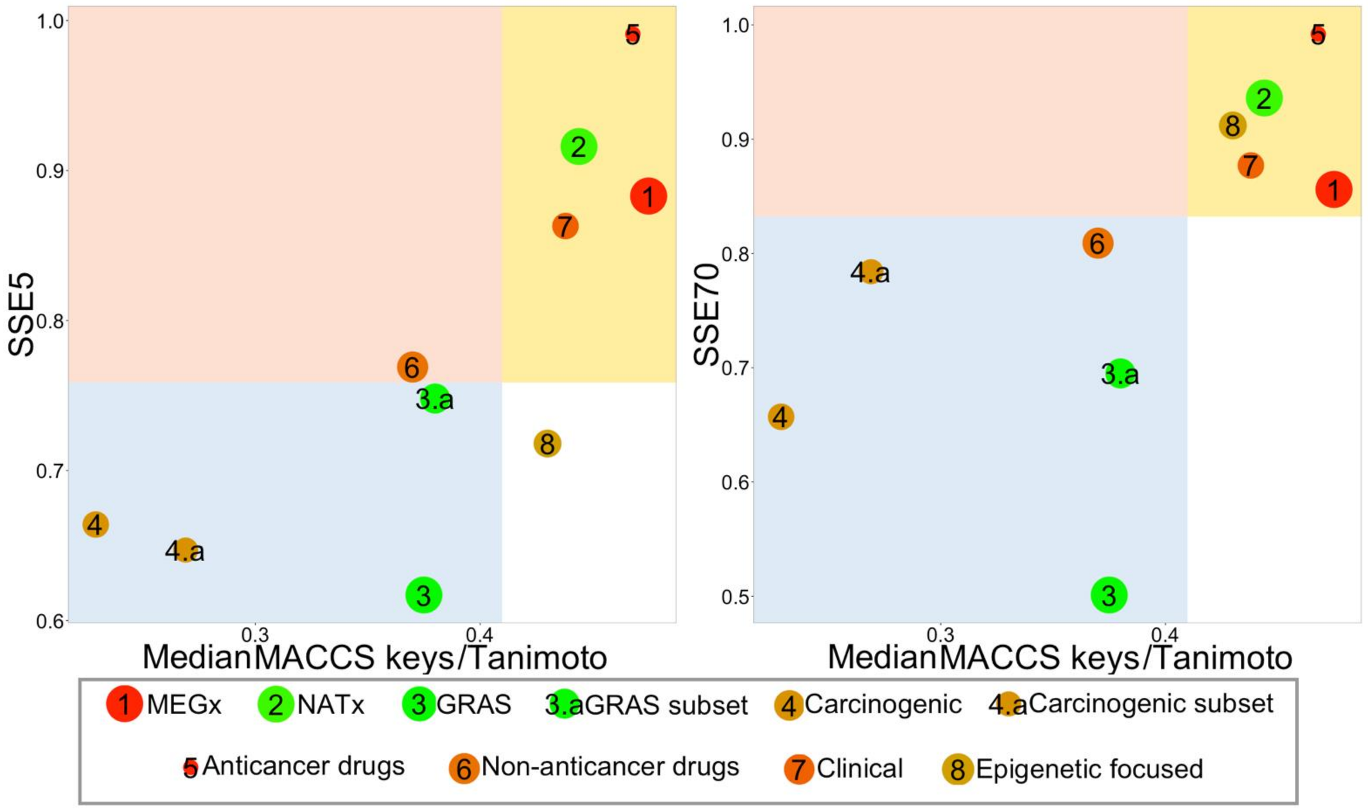

Figure S3. Consensus Diversity Plots using measures of scaffold diversity for the most populated scaffolds (SSE5 and SSE40). The median values of the distribution of SSE5 SSE40 and MACCS/Tanimoto similarity values of all the data sets were used to set the quadrants in the CDPs.
